# Association between triglyceride glucose-body mass index and obstructive sleep apnea: a study from NHANES 2015–2018

**DOI:** 10.3389/fnut.2024.1424881

**Published:** 2024-08-16

**Authors:** Xingru Meng, Haihua Wen, Leshen Lian

**Affiliations:** ^1^Department of Respiratory Medicine, Dongguan Hospital of Traditional Chinese Medicine, Dongguan, Guangdong, China; ^2^The Ninth Clinical Medical College, Guangzhou University of Chinese Medicine, Dongguan, Guangdong, China

**Keywords:** obstructive sleep apnea, triglyceride glucose-body mass index, insulin resistance, NHANES, cross-sectional study

## Abstract

**Background:**

The association between TyG-BMI index and the risk of obstructive sleep apnea (OSA), a recently identified biomarker indicating insulin resistance, has yet to be elucidated. Therefore, this study aimed to investigate the association between TyG-BMI index and the risk of OSA using the NHANES database.

**Methods:**

Analyses were performed on NHANES data conducted between 2015 and 2018. Logistic regression, stratified analyses, curve-fitting analyses, and threshold effects analyses were utilized to assess the association between TyG-BMI index and the risk of OSA.

**Results:**

The study included 4,588 participants. Multifactorial logistic regression analyses found a significant association between TyG-BMI and increased risk of OSA [OR: 1.54 (CI:1.39–1.70)]. In stratified analyses, age interacted with the association, with TyG-BMI being associated with increased risk of OSA only in a subgroup of subjects younger than 60 years [1.31 (1.14–1.50)], but gender, smoking status, and alcohol use, did not influence the association. The presence of diabetes, hypertension, and cardiovascular diseases also modified the association, but the number of the included subjects with such conditions was significantly lower, therefore the significance of associations was not observed in those subgroups. Additionally, the risk was non-linearly associated, with the inflection point of TyG-BMI at 12.09, after which the lower slope in the risk was observed.

**Conclusion:**

This study demonstrates that elevated levels of the TyG-BMI index are correlated with risk for OSA, underscoring the significance of these findings in facilitating early prevention or timely intervention for OSA.

## 1 Introduction

Obstructive sleep apnea syndrome (OSAS) presents a prevalent health concern characterized by the recurring occurrence of apnea and hypoventilation during sleep. Clinical manifestations typically encompass symptoms such as snoring, repeated awakenings from suffocation, daytime somnolence, and, in severe instances, cognitive deterioration or behavioral irregularities (1). Frequent apnea and hypoventilation at night in OSA lead to increased carbon dioxide and sympathetic arousal, as well as increased inflammatory and oxidative stress, resulting in insufficient antioxidant capacity. This situation can lead to or exacerbate cardiovascular and metabolic disorders (2, 3). According to statistics, nearly 1 billion adults worldwide may suffer from OSA, and the prevalence rate is as high as 50% in some countries, potentially increasing the prevalence rate of OSA globally with changes in lifestyles and the growth of the aging population (4).

The TyG-BMI index was composed of the fasting blood glucose index, the triglyceride (TG) index, and the BMI index. Numerous studies have additionally substantiated a noteworthy association between the TyG-BMI index and metabolic diseases (5), cardiovascular disease (6), and cerebrovascular disease (7). Subsequent investigation by Er et al. (8). uncovered that integrating body mass index (BMI) with the TyG index offers a more holistic approach to identifying individuals at elevated risk of visceral obesity, lipofactors, organic metabolic abnormalities, and cardiometabolism, surpassing the efficacy of solely relying on the TyG index.

Current research indicated a close association between OSA and cardiovascular, metabolic, and cerebrovascular diseases (9–11). The presence of risk factors such as obesity (12), alcoholism (13), smoking (14), hypertension (15), diabetes mellitus (16), hyperlipidemia (17), and the metabolic syndrome (18) was found to correspondingly increase the risk of OSA and related diseases (19, 20). For instance, obesity resulted in fat deposition in the upper respiratory tract, leading to OSA, which in turn exacerbated the metabolic dysfunction associated with obesity (21). Additionally, OSA could interact with related diseases through oxidative stress, inflammatory responses, sympathetic activation, and other abnormal mechanisms associated with cardiovascular or metabolic disorders (22). In turn, relevant cross-sectional studies have emphasized the bidirectional association of OSA with various adverse metabolic conditions, such as NAFLD, dyslipidemia, insulin resistance, and atherosclerosis (23). Given the bidirectional relationship between OSA and multiple diseases, early identification and management of OSA risk factors are crucial. The TyG-BMI index integrates the interaction between lipid metabolism, glucose homeostasis, and obesity (8). Together, these factors were pivotal in the development of OSA (24, 25). Among them, changes in glucose metabolism, particularly insulin resistance, were independently associated with OSA (26, 27). Previous studies have demonstrated that the TyG-BMI index can serve as a straightforward and robust alternative indicator for early detection of insulin resistance (8). Therefore, exploring the association between TyG-BMI and OSA is essential for OSA prevention and management. However, studies investigating this relationship are limited. Thus, this study aimed to investigate the association between TyG-BMI and OSA using the NHANES database.

## 2 Methods

### 2.1 Study sources

The NHANES (National Health and Nutrition Examination Survey) is a research initiative designed to evaluate the health and nutritional status of both adults and children in the United States. This database contains health and nutrition related data on a wide range of populations, and by collecting and analyzing the relevant data, researchers can gain insight into the prevalence and risk factors of diseases, thus serving as a foundation for the formulation of pertinent policies and interventions (8, 28). The study protocol received approval from the Research Ethics Review Board of the National Center for Health Statistics (NCHS). Data for 2015–2016, 2017–2018 were obtained from public databases.^1^

### 2.2 Study population

**Figure 1:**
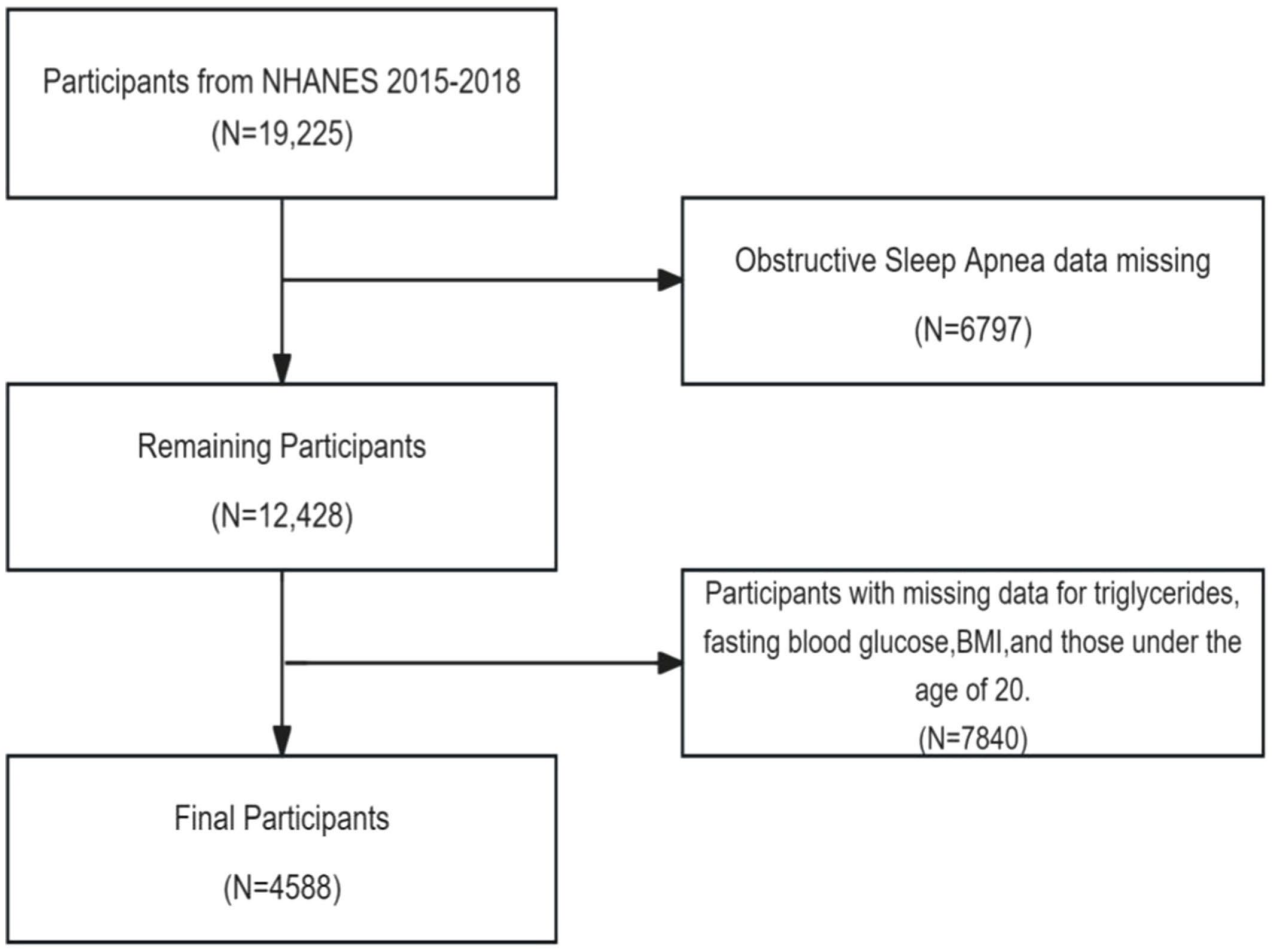
Flow chart of participant screening.

Data from NHANES 2015–2016 and 2017–2018 were used, which included a total of 19,225 participants. Participants with the following conditions were excluded in study: (1) missing questionnaire data related to OSA; (2) missing triglyceride data, fasting glucose data, and BMI data; (3) participants aged <20 years. The analysis involved the final set of 4,588 included subjects (Figure 1).

### 2.3 Assessment of the OSA

OSA status was determined based on questionnaire data concerning sleep habits and disorders (29). The patient answered “yes” or “no” to the following questions According to relevant studies, OSA was defined as the presence of any of the following: (1) snoring three or more times per week; (2) wheezing, snoring, or stopping breathing at least three nights per week; (3) feeling drowsy more than 16–30 times during the day, despite sleeping seven or more hours per night.

### 2.4 Assessment of the TyG-BMI index

The calculation of the TyG-BMI index was performed using the following formula (8).

TyG = Ln[fasting triglycerides (mg/dL) × fasting blood glucose (mg/dL)/2].

BMI = weight (kg)/height (m)^2^.

TyG-BMI = TyG index × BMI.

### 2.5 Study covariates

In this study, potential confounders comprised 10 covariates: age, gender, race, the ratio of family income to poverty (PIR), education level, smoking status and drinking status, hypertension, diabetes, and cardiovascular disease(CVD). Age was categorized as under 60 and over 60. Race was categorized as Mexican American, Non-Hispanic White, Non-Hispanic Black, and other races; and education level was categorized as less than high school, high school diploma, and high school and above. Smoking status was categorized based on lifetime smoking, with less than 100 cigarettes and more than 100 cigarettes as thresholds, and alcohol use was defined as never having consumed alcohol in the past year and ≥ 12 drinks/year. Hypertension was defined as being told by a doctor that one has hypertension. Diabetes was defined as being told by a doctor that one has diabetes. Cardiovascular disease was defined as being told by a doctor that one has congestive heart failure, coronary heart disease, angina, heart attack, or stroke. Those who answered “yes” were considered to have hypertension, diabetes, and cardiovascular disease. Health status was evaluated using self-reported and physician or other health professional assessments of hypertension, diabetes, and cardiovascular disease.

### 2.6 Statistical analysis

Demographic characteristics were evaluated utilizing the chi-square test and Kruskal-Wallis H-test. Categorical variables were presented as frequencies or percentages, Continuous variables were tested for normality using the Kolmogorov–Smirnov test, with non-normal distributions expressed as medians and quartiles, and normal distributions expressed as means and standard deviations. The multivariate model included gender, age, race, PIR, education level, smoking status and alcohol use, hypertension, diabetes, and CVD, with all of the above indicators serving as covariates. The TyG-BMI index was divided into quartiles, with the lowest quartile (Q1) serving as the reference group. Multiple logistic regression models were employed to investigate the association between the TyG-BMI index and OSA across three models. Model 1 remained unadjusted, while model 2 was adjusted for age, gender, and race. In model 3, various demographics including age, gender, race, education level, PIR, and pertinent health factors such as smoking status, alcohol use, hypertension, diabetes, and CVD were adjusted for. The variance inflation factor (VIF) measures the degree of multicollinearity. All regression models have a VIF of less than 4, indicating that there is no high degree of multicollinearity between the covariates of the regression model (Supplementary Table 1). Smoothed curve fitting using the generalized additive model (GAM) was utilized to explore the non-linear relationship between TyG-BMI and OSA. Additionally, Subgroup analyses were conducted using stratified multivariate regression analyses for gender, age, race, smoking status, alcohol use, hypertension, diabetes, and CVD. Interaction tests were employed to evaluate potential alterations in relationships among subgroups. Statistical analyses were carried out using R (version 3.4.3) and EmpowerStats (version 2.0). *p* < 0.05 was considered statistically significant.

## 3 Results

### 3.1 Baseline characteristics

The final sample comprised 4,588 adults, with 48.10% male and 52% female participants. The prevalence rate of OSA was 50.35%. The majority of participants were Non-Hispanic White. Statistical differences were observed in gender, age, PIR, education level, race, smoking status, alcohol use, CVD, hypertension, and diabetes status (*p* < 0.05). Several characteristics were highest in the highest quartile group, including BMI >30 kg/m^2^, age > 60 years, male gender, education levels below high school, smoking, and no alcohol consumption. Conversely, those with BMI <25 kg/m^2^, high school education or higher, and lower income showed the lowest prevalence in the highest quartile. Mexican Americans and other Hispanic populations exhibited the highest prevalence in the highest two quartiles, while Non-Hispanic Black and other racial groups had the lowest. The prevalence of diabetes, hypertension, and cardiovascular disease was significantly higher in the highest two quartiles compared to the lowest two (*p* < 0.05). The highest quartile group exhibited elevated levels of triglycerides, fasting glucose, and TyG index compared to the lowest quartile group (Table 1).

**Table 1:** Basic characteristics of participants in the TyG-BMI index quartiles.

Characteristics	TyG-BMI Index					
	Total	Q1	Q2	Q3	Q4	*p*-value
	(*N* = 4,588)	(*n* = 1,147)	(*n* = 1,147)	(*n* = 1,147)	(*n* = 1,147)	
TyG-BMI	11.88(11.35–12.42)	11.00 (10.73–11.19)	11.63 (11.50–11.76)	12.13 (12.00–12.29)	12.82 (12.59–13.18)	<0.001
Triglycerides(mg/dl)	4.53(4.12–4.92)	3.89(3.66–4.09)	4.37 (4.20–4.53)^***^	4.73 (4.56–4.91)^***^	5.18 (4.94–5.47)^***^	<0.001
Glucose(mg/dl)	4.63(4.56–4.74)	4.56(4.51–4.62)	4.61(4.55–4.69)^***^	4.66(4.59–4.74)^***^	4.77(4.65–5.06)^***^	<0.001
TyG	8.50(8.05–8.97)	7.77(7.54–7.97)	8.30(8.14–8.45)^***^	8.72(8.56–8.88)^***^	9.33(9.10–9.66)^***^	<0.001
BMI kg/m^2^	28.50(24.60–33.50)	23.70(21.40–26.90)	27.70(24.70–31.50)^***^	29.90(26.70–34.20)^***^	33.20(29.00–33.20)^***^	<0.001
BMI(%)			***	***	***	<0.001
>30	1848 (40.28%)	140 (12.21%)	367 (32.00%)	555 (48.39%)	786 (68.53%)	
>25, ≤30	1,488 (32.43%)	302 (26.33%)	462 (40.28%)	434 (37.84%)	290 (25.28%)	
≤25	1,252 (27.29%)	705 (61.46%)	318 (27.72%)	158 (13.78%)	71 (6.19%)	
Age(years)	52(36–65)	41(28–60)	53(36–65)^***^	53(39–66)^***^	56(43–65)^***^	<0.001
Age(%)			***	***	***	<0.001
<60	2,930 (63.86%)	853 (74.37%)	702 (61.20%)	697 (60.77%)	678 (59.11%)	
> = 60	1,658 (36.14%)	294 (25.63%)	445 (38.80%)	450 (39.23%)	469 (40.89%)	
PIR(%)	2.10(1.15–3.98)	2.24(1.24–4.17)	2.07(1.10–4.01)	2.11(1.14–3.79)	1.97(1.11–3.69)^*^	0.013
Gender(%)			*	**	***	<0.001
Male	2,207 (48.10%)	478 (41.67%)	545 (47.52%)	578 (50.39%)	606 (52.83%)	
Female	2,381 (51.90%)	669 (58.33%)	602 (52.48%)	569 (49.61%)	541 (47.17%)	
Race(%)			***	***	***	<0.001
Mexican American	708 (15.43%)	96 (8.37%)	176 (15.34%)	193 (16.83%)	243 (21.19%)	
Other Hispanic	536 (11.68%)	89 (7.76%)	120 (10.46%)	172 (15.00%)	155 (13.51%)	
Non-Hispanic White	1,551 (33.81%)	376 (32.78%)	372 (32.43%)	382 (33.30%)	421 (36.70%)	
Non-Hispanic Black	993 (21.64%)	344 (29.99%)	296 (25.81%)	201 (17.52%)	152 (13.25%)	
Other Race	800 (17.44%)	242 (21.10%)	183 (15.95%)	199 (17.35%)	176 (15.34%)	
Education level(%)			*	***	***	<0.001
Less than high school	1,005 (21.91%)	183 (15.97%)	239 (20.84%)	275 (23.98%)	308 (26.85%)	
High school	1,044 (22.76%)	249 (21.73%)	270 (23.54%)	264 (23.02%)	261 (22.76%)	
More than high school	2,538 (55.33%)	714 (62.30%)	638 (55.62%)	608 (53.01%)	578 (50.39%)	
Smoking status(%)				*	***	<0.001
No	2,592 (56.54%)	714 (62.30%)	679 (59.25%)	632 (55.20%)	567 (49.43%)	
Yes	1992 (43.46%)	432 (37.70%)	467 (40.75%)	513 (44.80%)	580 (50.57%)	
Alcohol use(%)			**		***	<0.001
No	825 (22.41%)	153 (17.13%)	215 (23.52%)	193 (20.60%)	264 (28.18%)	
Yes	2,856 (77.59%)	740 (82.87%)	699 (76.48%)	744 (79.40%)	673 (71.82%)	
Diabetes(%)			***	***	***	<0.001
No	3,706 (83.26%)	1,073 (95.55%)	1,007 (90.31%)	925 (84.09%)	701 (62.98%)	
Yes	745 (16.74%)	50 (4.45%)	108 (9.69%)	175 (15.91%)	412 (37.02%)	
Hypertension(%)			***	***	***	<0.001
No	2,851 (62.24%)	885 (77.23%)	743 (64.89%)	657 (57.38%)	566 (49.43%)	
Yes	1730 (37.76%)	261 (22.77%)	402 (35.11%)	488 (42.62%)	579 (50.57%)	
CVD(%)			***	***	***	<0.001
No	4,036 (87.97%)	1,074 (93.64%)	1,000 (87.18%)	1,013 (88.32%)	949 (82.74%)	
Yes	552 (12.03%)	73 (6.36%)	147 (12.82%)	134 (11.68%)	198 (17.26%)	
OSA(%)			***	***	***	<0.001
No	2,278 (49.65%)	716 (62.42%)	601 (52.40%)	522 (45.51%)	439 (38.27%)	
Yes	2,310 (50.35%)	431 (37.58%)	546 (47.60%)	625 (54.49%)	708 (61.73%)	

TyG-BMI, triglyceride glucose-body mass index; BMI, body mass index; TyG, triglyceride glucose; PIR, Poverty Income Ratio; CVD, Cardiovascular Disease; OSA, obstructive sleep apnea. Compared to the TyG-BMI Index Q1 group: *p < 0.05, **p < 0.01, ***p < 0.001.

### 3.2 Association between TyG-BMI index and risk for OSA

**Figure 2:**
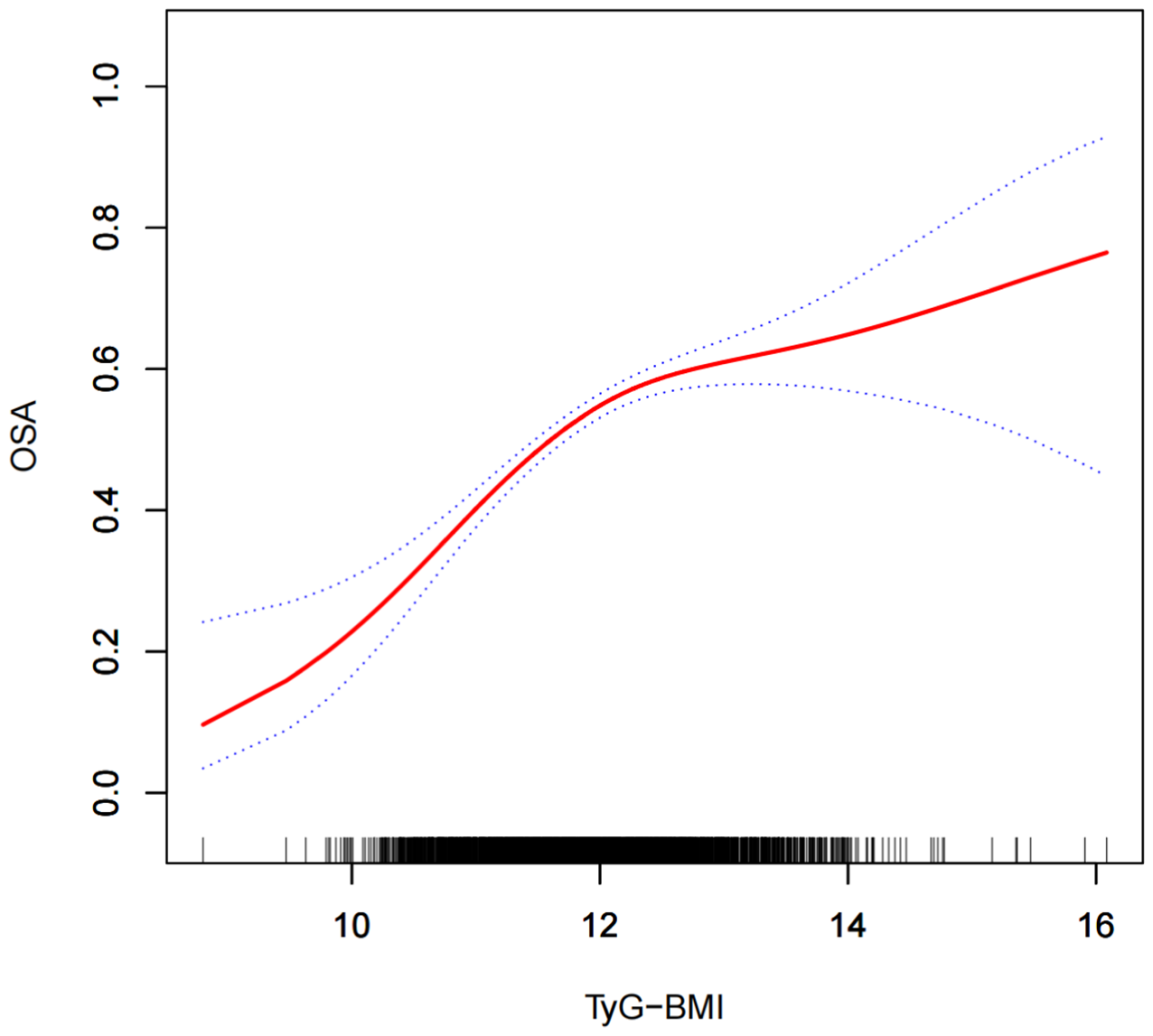
The solid red line illustrates the smooth curve fit between the variables, while the blue bands depict the 95% confidence interval derived from the fit.

Multifactorial logistic regression analyses revealed a significant positive association between TyG-BMI and the risk of OSA, which remained statistically significant across all models (*p* < 0.05). After adjusting for all covariables, each 1-unit increase in TyG-BMI was associated with a 54% increase in the prevalence of OSA among participants [1.54(1.39–1.70)]. Participants in the quartile with the highest TyG-BMI levels had a 136% increase in OSA prevalence compared to those in the quartile with the lowest TyG-BMI levels (Table 2). The smoothed curve fitting indicated a TyG-BMI index cut-off of 12.09 units, and indicated that when the TyG-BMI level was lower than 12.09, the prevalence of OSA increased by 85% for every 1-unit increase in TyG-BMI (*p* < 0.001), but after this inflection point, the association was more dispersed and the prevalence of OSA increased by 18% for every 1-unit increase in TyG-BMI (*p* = 0.058; Table 3). The 2,775 (60.48%) of subjects had TyG-BMI below this inflection point, while 1813(39.51%) of subjects had TyG-BMI above this inflection point (Figure 2).

**Table 2:** Association between TyG-BMI index and the risk of OSA.

Exposure	Model 1 OR(95%CI) *P*-value	Model 2 OR(95%CI) *p*-value	Model 3 OR(95%CI) *p*-value
TyG-BMI index	1.62 (1.50, 1.75) <0.0001	1.57 (1.45, 1.70) <0.0001	1.54 (1.39, 1.70) <0.0001
TyG-BMI index quartile			
Q1	Reference	Reference	Reference
Q2	1.51 (1.28, 1.78) <0.0001	1.42 (1.20, 1.68) <0.0001	1.50 (1.22, 1.85) 0.0001
Q3	1.99 (1.68, 2.35) <0.0001	1.87 (1.57, 2.22) <0.0001	2.00 (1.62, 2.47) <0.0001
Q4	2.68 (2.26, 3.17) <0.0001	2.49 (2.09, 2.97) <0.0001	2.36 (1.89, 2.94) <0.0001
*P* for trend	<0.0001	<0.0001	<0.0001

TyG-BMI, triglyceride glucose-body mass index; OSA, obstructive sleep apnea. Model 1: non-adjusted. Model 2: adjusted for age, gender, and race. Model 3: adjusted for age, gender, race, education level, PIR, smoking status, drinking status, hypertension, diabetes, CVD.

**Table 3:** Analysis of the threshold effect between TyG-BMI index and the risk of OSA.

Threshold effect analysis	OSA OR (95%CI) *p*-value
TyG-BMI	
Inflection point of TyG-BMI (K)	12.09
<K slope n = 2,775(60.48%)	1.85 (1.60, 2.14) 0.0001
>K slope n = 1813(39.51%)	1.18 (0.99, 1.39) 0.0576
Log-likelihood ratio test	<0.001

TyG-BMI, triglyceride glucose-body mass index; OSA, obstructive sleep apnea.

### 3.3 Stratified analyses

In stratified analyses, performed using TyG-BMI as continuous variable and applying the model 3, age significantly interacted with the association, with TyG-BMI being associated with increased risk of OSA only in a subgroup of subjects younger than 60 years [1.31 (1.14–1.50)], but gender, smoking status, alcohol use and race did not influence the association. The presence of diabetes, hypertension, and cardiovascular diseases also modified the association, but the number of the included subjects with such conditions was significantly lower, therefore the significance of associations was not observed in those subgroups (Table 4).

**Table 4:** Stratified analysis of the association between TyG-BMI and the risk of OSA.

Subgroup	OR(95%CI)	*P* for interaction
Gender		0.2749
Male	1.47 (1.29, 1.68) <0.0001	
Female	1.63 (1.41, 1.88) <0.0001	
Race		0.9466
Mexican American	1.61 (1.23, 2.12) 0.0006	
Other Hispanic	1.43 (1.04, 1.97) 0.0267	
Non-Hispanic White	1.52 (1.29, 1.78) <0.0001	
Non-Hispanic Black	1.65 (1.32, 2.07) <0.0001	
Other Race	1.59 (1.22, 2.06) 0.0005	
Age		0.0027
>60	0.89 (0.71, 1.11) 0.2879	
<=60	1.31 (1.14, 1.50) <0.0001	
Smoking status		0.3833
No	1.48 (1.29, 1.69) <0.0001	
Yes	1.60 (1.40, 1.84) <0.0001	
Alcohol use		0.9273
No	1.53 (1.24, 1.87) <0.0001	
Yes	1.54 (1.38, 1.72) <0.0001	
Diabetes		0.0145
No	1.63 (1.46, 1.83) <0.0001	
Yes	1.19 (0.95, 1.49) 0.1262	
Hypertension		0.0015
No	1.73 (1.53, 1.97) <0.0001	
Yes	1.24 (1.05, 1.46) 0.0128	
CVD		0.0079
No	1.61 (1.44, 1.80) <0.0001	
Yes	1.05 (0.78, 1.41) 0.7346	

TyG-BMI, triglyceride glucose-body mass index; CVD, cardiovascular disease; OSA, obstructive sleep apnea. Adjusted for age, gender, race, education level, PIR, smoking status, alcohol use, hypertension, diabetes.

## 4 Discussion

In this cross-sectional study, involving 4,588 adults, a positive association between high TyG-BMI index and the risk of OSA was observed. This association persisted even after adjusting for all covariates. The findings indicated a 54% rise in OSA prevalence for each 1-unit increase in TyG-BMI [OR: 1.54, 95% CI: 1.39–1.70]. The highest TyG-BMI quartile was associated with 2.36 higher odds for OSA compared with the lowest quartile. In stratified analyses, TyG-BMI was associated with an increased risk of OSA only in the subgroup of subjects younger than 60 years of age [1.31 (1.14–1.50)]. The results of the threshold effect analysis showed that when the TyG-BMI level was lower than 12.09, the prevalence of OSA increased by 85% for every 1-unit increase in TyG-BMI (*p* < 0.001), but after this inflection point, the association was more dispersed and the prevalence of OSA increased by 18% for every 1-unit increase in TyG-BMI (*p* = 0.058).

TyG-BMI was found to be a superior predictor of metabolic diseases such as diabetes, cardiovascular disease, and prognosis (30–32). It offers a more comprehensive assessment of metabolic health compared to individual biomarkers, as the interactions among lipid metabolism, glucose homeostasis, and obesity were considered (8). TyG-BMI simultaneously captured BMI, blood glucose, and lipid levels. It exhibited high predictive capability for insulin resistance (33). Numerous studies have demonstrated strong associations between risk factors such as obesity, hypertension, diabetes mellitus, hyperlipidemia, and metabolic syndrome, as well as poor lifestyle habits including alcoholism and smoking, with OSA (19). It has been reported that over 40% of individuals with a BMI over 30 had obstructive sleep apnea, and 60% of patients diagnosed with metabolic syndrome were found to have OSA (34). For example, obesity could result in fat deposition in the upper respiratory tract or induce systemic inflammation, leading to OSA. Conversely, OSA could exacerbate metabolic, inflammatory, vascular, and cardiac dysfunctions caused by obesity, thereby worsening the metabolic syndrome and creating a vicious cycle (21). Additionally, varying degrees of OSA have been observed in patients with hyperlipidemia, hypertension, and diabetes (15, 35). The sleep disorder and intermittent hypoxia characteristic of OSA have been shown to trigger sympathetic excitation and inflammation, as well as cause vascular endothelial damage, altered coagulation function, abnormal lipid metabolism, and disruptions in glucose homeostasis (36–38). These mechanisms may exacerbate existing metabolic or cardiovascular diseases, contributing to a vicious cycle (9, 39). Cohort studies indicated that individuals diagnosed with OSA faced a markedly heightened risk of future coronary heart disease events and diabetes (40). Cross-sectional studies further underscored OSA as an independent risk factor for several adverse metabolic conditions, including NAFLD, dyslipidemia, insulin resistance, and atherosclerosis (23).

Lipid metabolism, glucose homeostasis, and obesity collectively played a pivotal role in the development of OSA (24, 25). Particularly noteworthy was that changes in glucose metabolism and OSA were independently linked (27). It has been demonstrated that respiratory disturbances in rats with hyperglycemia induced by streptozotocin improved with the use of antidiabetic drugs. This study suggests that glycemic changes cannot be ignored in the development of OSA (41, 42). Conversely, OSA disrupts the dynamic balance of blood glucose. Animal studies have indicated that intermittent hypoxia leads to pancreatic β-cell dysfunction and insulin resistance in insulin-sensitive organs and adipose tissue (43). Additionally, the high levels of inflammatory factors found in the upper and lower airways of hypoxic mice, coupled with the vicious cycle between OSA and airway inflammation, ultimately contributed to insulin resistance (44, 45). Other studies have also confirmed that the hypoxic state of OSA was preferentially activated the pro-inflammatory factor NF-κB-mediated pathway, possibly caused by an inflammatory response to hypoxic exposure via adipocytes (46). This inflammatory change, in turn, was accompanied by an increasing polarization of pro-inflammatory M1 macrophages, expression of inducible nitric oxide synthase, and changes in the severity of insulin resistance, a mechanism that may have been a key link between OSA and the development of Insulin resistance (47). In turn, this inflammatory response mechanism was central to the pathogenesis of OSA-related cardiometabolic processes, which were ultimately led to the development of cardiovascular disease, resulting in a vicious circle (48, 49). In addition, it was found that the hypoxic stress of OSA activated the hypothalamic–pituitary–adrenal axis, resulting in elevated cortisol levels and ultimately leading to insulin resistance. Studies on intermittent hypoxia in rodents confirmed this mechanism (50). In another animal study, intermittent hypoxia was demonstrated to mediate the expression of hypoxia-inducible factor 1αin pancreatic β-cells. This led to increased reactive oxygen species and ultimately resulted in insulin resistance (51). This hypoxic stress mechanism also contributed to the development of other diseases that induced insulin resistance through oxidative stress and islet cell apoptosis via TRB3 and p-JNK pathways, thereby contributing to the development of type 2 diabetes mellitus in OSA populations (52). In the characteristic sleep deprivation and sleep fragmentation of OSA, sympathetic activation was induced, negatively affecting insulin secretion and sensitivity. This was manifested as increased blood pressure, reduced heart rate variability, and diminished sensitivity of sympathetic reflexes (53, 54). These phenomena were observed to improve after short-term CPAP treatment (55). Restricted sleep states in OSA were observed to result in a sudden and massive secretion of growth hormone just before sleep onset, which persisted during the night and had a negative effect on glucose regulation (56, 57).

In a study involving women with polycystic ovary syndrome (PCOS), a condition linked to insulin resistance, researchers frequently observed sleep apnea and daytime sleepiness. Subsequent investigations revealed that insulin resistance emerged as a superior predictor of sleep apnea compared to testosterone levels, age, and BMI (58). Sanapo et al. reported similar findings. After adjusting for factors influencing glucose metabolism, the onset of OSA was observed to correlate with an early rise in insulin resistance, demonstrating a dose–response relationship between severity and insulin resistance (59). The underlying mechanism behind this association could be attributed to insulin resistance induced fat deposition in the airway tissue, leading to compromised pharyngeal collapsibility. This heightened risk of airway obstruction subsequently increases susceptibility to OSA. Moreover, even in the absence of sleep apnea, insulin resistance remains correlated with impaired mechanical function of the upper airway (60). Furthermore, patients with OSA exhibit higher ratios and areas of visceral fat to total fat (61). Insulin resistance that occurred in adipose tissue elevated levels of free fatty acids, causing or exacerbating visceral fat accumulation (62). Alterations in this mechanism may have led to the worsening or occurrence of OSA. Visceral adiposity resulting from insulin resistance led to an imbalance in the secretion of IL-6 and CRP inflammatory mediators. The resultant systemic low inflammatory state has been demonstrated as a risk factor in the pathogenesis of OSA (63, 64). The above mechanistic studies suggest that the link between OSA and IR may have been established through mechanisms such as sleep fragmentation, sympathetic arousal, intermittent hypoxia and inflammation, with significant implications for the development of a variety of diseases. In this study, a significant correlation between OSA and insulin resistance was observed. Once again, the importance of emphasizing insulin resistance in OSA is underscored, along with the need to mitigate potential risk factors for the progression of OSA to other diseases.

Our findings also indicated that even in the fully adjusted model, a positive association persisted between the two, underscoring the significance of insulin resistance in OSA. Moreover, TyG-BMI index exhibited high sensitivity and specificity in identifying insulin resistance, suggesting that monitoring the TyG-BMI index could aid healthcare providers in identifying individuals at risk of OSA earlier and implementing appropriate interventions. In addition, stratified analyses indicated no association between individuals over 60 years of age, those with hypertension, diabetes, cardiovascular disease, and OSA. We considered the possibility that OSA was inherently more common in a variety of cardiovascular as well as metabolic disorders (65). Secondly, in subjects with diabetes mellitus, hypertension, and cardiovascular diseases, there may have been lipid-lowering and glucose-lowering medications that could have had an impact on the TyG-BMI index (66). The absence of association in people over 60 years of age could have been related to age-related metabolic and physiological changes, such as alterations in lipid storage patterns and associated metabolic markers (67). With age, older adults typically experienced elongation of the upper airway, reduction in the size of the hyoid bone, and decreased responsiveness of hyoid muscles to negative pressure. Thus, it appeared that the occurrence of sleep apnea in older adults might have been more related to structural and functional changes in the upper airway (68, 69). Therefore, the necessity for close monitoring and analysis to identify changes in TyG-BMI levels to mitigate the incidence of OSA was even more pronounced in individuals younger than 60 years of age and without metabolic or cardiovascular disease than in the aforementioned populations.

To compare the predictive value for OSA of the TyG-BMI index with the predictive value of TyG index alone and BMI alone, we also performed the supplementary regression analyses (Supplementary Tables 2, 3). We found that the highest quartile of BMI was associated with a 3.42-fold increased likelihood of OSA compared to the lowest quartile (Supplementary Table 2). This finding suggests that the TyG-BMI index did not demonstrate superior predictive value for OSA compared to BMI alone. Obesity was widely recognized as a acknowledged risk factor for OSA (70). Research has demonstrated that a 32% increase in apnoea hypoventilation index (AHI) was implied by every 10% rise in body weight (71). It was observed by Akanbi et al. that the risk of OSA increased with increasing BMI, and the ratio of OSA risk was as high as 15.76(95%Cl7.44–33.9) when BMI exceeded 35 kg/m^2^ (72). This was consistent with our findings. However, it has been suggested that BMI should not be the sole metric considered, as it does not account for the distribution of body fat, differences in neck circumference, waist-to-hip ratio, and cephalometric measurements of the tongue and oropharyngeal region (73–75). For instance, in Asian populations, anatomical differences in the head and face based on racial traits may contribute to OSA among non-obese individuals (76). To further evaluate the predictive capacity of the TyG-BMI index, we similarly analyzed the TyG index, revealing that the highest quartile of TyG levels increased the prevalence of OSA by 1.26-fold (Supplementary Table 3). However, the predictive value of the TyG index was slightly inferior compared with TyG-BMI [OR: 2.36 (CI: 1.89–2.94)].

To our knowledge, this study was the first to assess the association between TyG-BMI index and the risk of OSA. We used a large sample of the US population from the NHANES database and conducted cross-sectional analyses. Firstly, confounders were thoroughly adjusted to enhance the reliability of the findings. Secondly, the large sample size facilitated stratified analyses, thereby strengthening the study’s reliability. The results of the study indicated indicated a positive correlation between the TyG-BMI index and risk of OSA, suggesting an association between metabolic health and OSA. However, there were several limitations in our study. Firstly, cross-sectional studies could not capture dynamic changes in the index to establish causality. Moreover, the diagnosis of OSA was typically relied on polysomnography (PSG), which required specialized instruments for implementation and analysis by professionals. In contrast, questionnaires could be employed in diverse settings to initially screen individuals for OSA. Nevertheless, they may introduce recall bias and inaccuracies compared to objective sleep activity recordings (77). For example, some patients with moderate to severe OSA did not exhibit noticeable symptoms and may have been overlooked or excluded from the study, potentially leading to an underestimation of the high prevalence of OSA (78). Additionally, despite collecting fasting blood samples, individuals with comorbidities or other underlying conditions may have been taking medications that could impact TyG-BMI index (66). Another potentially more likely reason was that the number of participants included with hypertension, especially those with diabetes and cardiovascular disease, was too small to achieve statistical significance. However, we did not analyze the impact of medication on the association between TyG-BMI index and the risk of OSA, which could potentially affect the reliability of the results. Finally, our supplementary results indicated that the TyG-BMI index might have been less effective than BMI in predicting the risk of OSA, but more effective than the TyG index. We hypothesized that this could be due to the association of both TyG and BMI indices with obesity and metabolic health (79–81). There may have been overlap in their information, which could have potentially limited the enhancement of predictive power when used together. Additionally, various factors were associated with the occurrence of OSA, such as neck circumference, waist-to-hip ratio, family history, and others (73, 82). These factors could potentially influence the TyG-BMI index. Therefore, further studies are necessary to confirm the precise relationship between the TyG-BMI index and the risk of OSA, as well as to comprehensively compare relevant indicators and explore their potential application in clinical practice to enhance the prevention and treatment of metabolic health issues linked to OSA.

## 5 Conclusion

In conclusion, by using the data from NHANES surveys, this study revealed a positive association between TyG-BMI index and risk for OSA, particularly in the subgroup of subjects younger than 60 years. The association seems to have a higher slope below the inflection point of the TyG-BMI value of 12.09, and then becomes more dispersed. Because the cross-sectional study could not prove causality, to explore the mechanisms underlying the positive association between the TyG-BMI index and OSA, further studies are needed.

## Footnotes

^1^https://www.cdc.gov/nchs/nhanes/index.htm

## References

[1] Lv R Liu X Zhang Y Dong N Wang X He Y. Pathophysiological mechanisms and therapeutic approaches in obstructive sleep apnea syndrome. Signal Transduct Target Ther. (2023) 8:218. doi: 10.1038/s41392-023-01496-3, PMID: 37230968 PMC10211313

[2] Jehan S Myers AK Zizi F Pandi-Perumal SR Jean-Louis G McFarlane SI. Obesity, obstructive sleep apnea and type 2 diabetes mellitus: epidemiology and pathophysiologic insights. Sleep Med Disord. (2018) 2:52–8. doi: 10.15406/smdij.2018.02.00045 30167574 PMC6112821

[3] Kirk J Wickwire EM Somers VK Johnson DA Albrecht JS. Undiagnosed obstructive sleep apnea increases risk of hospitalization among a racially diverse group of older adults with comorbid cardiovascular disease. J Clin Sleep Med. (2023) 19:1175–81. doi: 10.5664/jcsm.10526, PMID: 36803353 PMC10315599

[4] Benjafield AV Ayas NT Eastwood PR Heinzer R Ip MSM Morrell MJ. Estimation of the global prevalence and burden of obstructive sleep apnoea: a literature-based analysis. Lancet Respir Med. (2019) 7:687–98. doi: 10.1016/S2213-2600(19)30198-5, PMID: 31300334 PMC7007763

[5] Hu H Han Y Cao C He Y. The triglyceride glucose-body mass index: a non-invasive index that identifies non-alcoholic fatty liver disease in the general Japanese population. J Transl Med. (2022) 20:398. doi: 10.1186/s12967-022-03611-4, PMID: 36064712 PMC9446832

[6] Nikbakht HR Najafi F Shakiba E Darbandi M Navabi J Pasdar Y. Triglyceride glucose-body mass index and hypertension risk in iranian adults: a population-based study. BMC Endocr Disord. (2023) 23:156. doi: 10.1186/s12902-023-01411-5, PMID: 37479987 PMC10360216

[7] Shao Y Hu H Li Q Cao C Liu D Han Y. Link between triglyceride-glucose-body mass index and future stroke risk in middle-aged and elderly chinese: a nationwide prospective cohort study. Cardiovasc Diabetol. (2024) 23:81. doi: 10.1186/s12933-024-02165-7, PMID: 38402161 PMC10893757

[8] Er LK Wu S Chou HH Hsu LA Teng MS Sun YC. Triglyceride glucose-body mass index is a simple and clinically useful surrogate marker for insulin resistance in nondiabetic individuals. PLoS One. (2016) 11:e0149731. doi: 10.1371/journal.pone.0149731, PMID: 26930652 PMC4773118

[9] Li YE Ren J. Association between obstructive sleep apnea and cardiovascular diseases. Acta Biochim Biophys Sin Shanghai. (2022) 54:882–92. doi: 10.3724/abbs.2022084, PMID: 35838200 PMC9828315

[10] Kawada T Otsuka T Nakamura T Kon Y. Relationship between sleep-disordered breathing and metabolic syndrome after adjustment with cardiovascular risk factors. Diabetes Metab Syndr. (2016) 10:92–5. doi: 10.1016/j.dsx.2015.10.005, PMID: 26545634

[11] Yaggi H Mohsenin V. Obstructive sleep apnoea and stroke. Lancet Neurol. (2004) 3:333–42. doi: 10.1016/S1474-4422(04)00766-5 15157848

[12] Punjabi NM. The epidemiology of adult obstructive sleep apnea. Proc Am Thorac Soc. (2008) 5:136–43. doi: 10.1513/pats.200709-155MG, PMID: 18250205 PMC2645248

[13] Simou E Britton J Leonardi-Bee J. Alcohol and the risk of sleep apnoea: a systematic review and meta-analysis. Sleep Med. (2018) 42:38–46. doi: 10.1016/j.sleep.2017.12.005, PMID: 29458744 PMC5840512

[14] Chang CW Chang CH Chuang HY Cheng HY Lin CI Chen HT. What is the association between secondhand smoke (SHS) and possible obstructive sleep apnea: a meta-analysis. Environ Health. (2022) 21:58. doi: 10.1186/s12940-022-00868-6, PMID: 35710478 PMC9202174

[15] Torres G Sánchez-de-la-Torre M Barbé F. Relationship between OSA and hypertension. Chest. (2015) 148:824–32. doi: 10.1378/chest.15-0136 25879807

[16] Aurora RN Punjabi NM. Obstructive sleep apnoea and type 2 diabetes mellitus: a bidirectional association. Lancet Respir Med. (2013) 1:329–38. doi: 10.1016/S2213-2600(13)70039-0, PMID: 24429158

[17] Barros D García-Río F. Obstructive sleep apnea and dyslipidemia: from animal models to clinical evidence. Sleep. (2019) 42:zsy236. doi: 10.1093/sleep/zsy236 30476296

[18] Castaneda A Jauregui-Maldonado E Ratnani I Varon J Surani S. Correlation between metabolic syndrome and sleep apnea. World J Diabetes. (2018) 9:66–71. doi: 10.4239/wjd.v9.i4.66, PMID: 29765510 PMC5951892

[19] Mitra AK Bhuiyan AR Jones EA. Association and risk factors for obstructive sleep apnea and cardiovascular diseases: a systematic review. Diseases. (2021) 9:88. doi: 10.3390/diseases9040088 34940026 PMC8700568

[20] Xiong MQ Hu WH Hu K Zheng ZS Dong ML Mo HH. Analysis of risk factors and consequences for concurrent obstructive sleep apnea in chronic obstructive pulmonary disease patients. Zhonghua Jie He He Hu Xi Za Zhi. (2019) 42:832–7. doi: 10.3760/cma.j.issn.1001-0939.2019.11.009, PMID: 31694093

[21] Patel SR Larkin EK Redline S. Shared genetic basis for obstructive sleep apnea and adiposity measures. Int J Obes. (2008) 32:795–800. doi: 10.1038/sj.ijo.0803803, PMID: 18209735 PMC2672200

[22] Jean-Louis G Zizi F Clark LT Brown CD McFarlane SI. Obstructive sleep apnea and cardiovascular disease: role of the metabolic syndrome and its components. J Clin Sleep Med. (2008) 4:261–72. doi: 10.5664/jcsm.27191 18595441 PMC2546461

[23] Framnes SN Arble DM. The bidirectional relationship between obstructive sleep apnea and metabolic disease. Front Endocrinol (Lausanne). (2018) 9:440. doi: 10.3389/fendo.2018.00440, PMID: 30127766 PMC6087747

[24] Gündüz C Basoglu OK Hedner J Zou D Bonsignore MR Hein H. Obstructive sleep apnoea independently predicts lipid levels: data from the European sleep apnea database. Respirology. (2018) 23:1180–9. doi: 10.1111/resp.13372, PMID: 30133061

[25] Huang T Sands SA Stampfer MJ Tworoger SS Hu FB Redline S. Insulin resistance, hyperglycemia, and risk of developing obstructive sleep apnea in men and women in the United States. Ann Am Thorac Soc. (2022) 19:1740–9. doi: 10.1513/AnnalsATS.202111-1260OC, PMID: 35385367 PMC9528746

[26] Ip MS Lam B Ng MM Lam WK Tsang KW Lam KS. Obstructive sleep apnea is independently associated with insulin resistance. Am J Respir Crit Care Med. (2002) 165:670–6. doi: 10.1164/ajrccm.165.5.2103001 11874812

[27] Punjabi NM Beamer BA. Alterations in glucose disposal in sleep-disordered breathing. Am J Respir Crit Care Med. (2009) 179:235–40. doi: 10.1164/rccm.200809-1392OC, PMID: 19011148 PMC2633056

[28] Zipf G Chiappa M Porter KS Ostchega Y Lewis BG Dostal J. National health and nutrition examination survey: plan and operations, 1999-2010. Vital Health Stat 1. (2013) 56:1–37. 25078429

[29] Gu X Tang D Xuan Y Shen Y Lu LQ. Association between obstructive sleep apnea symptoms and gout in US population, a cross-sectional study. Sci Rep. (2023) 13:10192. doi: 10.1038/s41598-023-36755-4, PMID: 37353548 PMC10290056

[30] Yang S Shi X Liu W Wang Z Li R Xu X. Association between triglyceride glucose-body mass index and heart failure in subjects with diabetes mellitus or prediabetes mellitus: a cross-sectional study. Front Endocrinol (Lausanne). (2023) 14:1294909. doi: 10.3389/fendo.2023.1294909 38027163 PMC10655238

[31] Peng N Kuang M Peng Y Yu H Zhang S Xie G. Associations between TyG-BMI and normal-high blood pressure values and hypertension: cross-sectional evidence from a non-diabetic population. Front Cardiovasc Med. (2023) 10:1129112. doi: 10.3389/fcvm.2023.1129112, PMID: 37168658 PMC10164981

[32] Huo RR Zhai L Liao Q You XM. Changes in the triglyceride glucose-body mass index estimate the risk of stroke in middle-aged and older Chinese adults: a nationwide prospective cohort study. Cardiovasc Diabetol. (2023) 22:254. doi: 10.1186/s12933-023-01983-5, PMID: 37716947 PMC10505325

[33] Lim J Kim J Koo SH Kwon GC. Comparison of triglyceride glucose index, and related parameters to predict insulin resistance in Korean adults: an analysis of the 2007-2010 Korean National Health and nutrition examination survey. PLoS One. (2019) 14:e0212963. doi: 10.1371/journal.pone.0212963, PMID: 30845237 PMC6405083

[34] Drager LF Togeiro SM Polotsky VY Lorenzi-Filho G. Obstructive sleep apnea: a cardiometabolic risk in obesity and the metabolic syndrome. J Am Coll Cardiol. (2013) 62:569–76. doi: 10.1016/j.jacc.2013.05.045, PMID: 23770180 PMC4461232

[35] Meszaros M Bikov A. Obstructive sleep Apnoea and lipid metabolism: the summary of evidence and future perspectives in the pathophysiology of OSA-associated Dyslipidaemia. Biomedicine. (2022) 10:2754. doi: 10.3390/biomedicines10112754, PMID: 36359273 PMC9687681

[36] McNicholas WT Bonsigore MR. Sleep apnoea as an independent risk factor for cardiovascular disease: current evidence, basic mechanisms and research priorities. Eur Respir J. (2007) 29:156–78. doi: 10.1183/09031936.00027406, PMID: 17197482

[37] Kent BD Grote L Ryan S Pépin JL Bonsignore MR Tkacova R. Diabetes mellitus prevalence and control in sleep-disordered breathing: the European sleep apnea cohort (ESADA) study. Chest. (2014) 146:982–90. doi: 10.1378/chest.13-2403 24831859

[38] Dopp JM Reichmuth KJ Morgan BJ. Obstructive sleep apnea and hypertension: mechanisms, evaluation, and management. Curr Hypertens Rep. (2007) 9:529–34. doi: 10.1007/s11906-007-0095-2, PMID: 18367017

[39] Giampá S Lorenzi-Filho G Drager LF. Obstructive sleep apnea and metabolic syndrome. Obesity (Silver Spring). (2023) 31:900–11. doi: 10.1002/oby.23679 36863747

[40] Strausz S Havulinna AS Tuomi T Bachour A Groop L Mäkitie A. Obstructive sleep apnoea and the risk for coronary heart disease and type 2 diabetes: a longitudinal population-based study in Finland. BMJ Open. (2018) 8:e022752. doi: 10.1136/bmjopen-2018-022752, PMID: 30327404 PMC6194468

[41] Hein MS Schlenker EH Patel KP. Altered control of ventilation in streptozotocin-induced diabetic rats. Proc Soc Exp Biol Med. (1994) 207:213–9. doi: 10.3181/00379727-207-43809, PMID: 7938052

[42] Ramadan W Petitjean M Loos N Geloen A Vardon G Delanaud S. Effect of high-fat diet and metformin treatment on ventilation and sleep apnea in non-obese rats. Respir Physiol Neurobiol. (2006) 150:52–65. doi: 10.1016/j.resp.2005.02.011, PMID: 16448934

[43] Ryan S. Adipose tissue inflammation by intermittent hypoxia: mechanistic link between obstructive sleep apnoea and metabolic dysfunction. J Physiol. (2017) 595:2423–30. doi: 10.1113/JP273312, PMID: 27901270 PMC5390885

[44] Lee EJ Heo W Kim JY Kim H Kang MJ Kim BR. Alteration of inflammatory mediators in the upper and lower airways under chronic intermittent hypoxia: preliminary animal study. Mediat Inflamm. (2017) 2017:1–7. doi: 10.1155/2017/4327237 PMC5606044 29038619

[45] Wieser V Moschen AR Tilg H. Inflammation, cytokines and insulin resistance: a clinical perspective. Arch Immunol Ther Exp. (2013) 61:119–25. doi: 10.1007/s00005-012-0210-1 23307037

[46] Taylor CT Kent BD Crinion SJ McNicholas WT Ryan S. Human adipocytes are highly sensitive to intermittent hypoxia induced NF-kappaB activity and subsequent inflammatory gene expression. Biochem Biophys Res Commun. (2014) 447:660–5. doi: 10.1016/j.bbrc.2014.04.062, PMID: 24755071

[47] Murphy AM Thomas A Crinion SJ Kent BD Tambuwala MM Fabre A. Intermittent hypoxia in obstructive sleep apnoea mediates insulin resistance through adipose tissue inflammation. Eur Respir J. (2017) 49:1601731. doi: 10.1183/13993003.01731-2016 28424360

[48] Arnaud C Poulain L Lévy P Dematteis M. Inflammation contributes to the atherogenic role of intermittent hypoxia in apolipoprotein-E knock out mice. Atherosclerosis. (2011) 219:425–31. doi: 10.1016/j.atherosclerosis.2011.07.122 21917260

[49] Ryan S Taylor CT McNicholas WT. Systemic inflammation: a key factor in the pathogenesis of cardiovascular complications in obstructive sleep apnoea syndrome. Thorax. (2009) 64:631–6. doi: 10.1136/thx.2008.105577, PMID: 19561283

[50] Dempsey JA Veasey SC Morgan BJ O'Donnell CP. Pathophysiology of sleep apnea. Physiol Rev. (2010) 90:47–112. doi: 10.1152/physrev.00043.2008, PMID: 20086074 PMC3970937

[51] Prabhakar NR Peng YJ Nanduri J. Hypoxia-inducible factors and obstructive sleep apnea. J Clin Invest. (2020) 130:5042–51. doi: 10.1172/JCI137560, PMID: 32730232 PMC7524484

[52] Zeng S Wang Y Ai L Huang L Liu Z He C. Chronic intermittent hypoxia-induced oxidative stress activates TRB3 and phosphorylated JNK to mediate insulin resistance and cell apoptosis in the pancreas. Clin Exp Pharmacol Physiol. (2024) 51:e13843. doi: 10.1111/1440-1681.13843 38302075

[53] Stamatakis KA Punjabi NM. Effects of sleep fragmentation on glucose metabolism in normal subjects. Chest. (2010) 137:95–101. doi: 10.1378/chest.09-0791, PMID: 19542260 PMC2803120

[54] Seravalle G Grassi G. Sleep apnea and hypertension. High Blood Press Cardiovasc Prev. (2022) 29:23–31. doi: 10.1007/s40292-021-00484-4 34739711

[55] Kent BD McNicholas WT Ryan S. Insulin resistance, glucose intolerance and diabetes mellitus in obstructive sleep apnoea. J Thorac Dis. (2015) 7:1343–57. doi: 10.3978/j.issn.2072-1439.2015.08.11, PMID: 26380761 PMC4561252

[56] Leproult R Van Cauter E. Role of sleep and sleep loss in hormonal release and metabolism. Endocr Dev. (2010) 17:11–21. doi: 10.1159/000262524, PMID: 19955752 PMC3065172

[57] Mullington JM Haack M Toth M Serrador JM Meier-Ewert HK. Cardiovascular, inflammatory, and metabolic consequences of sleep deprivation. Prog Cardiovasc Dis. (2009) 51:294–302. doi: 10.1016/j.pcad.2008.10.003, PMID: 19110131 PMC3403737

[58] Vgontzas AN Bixler EO Chrousos GP. Metabolic disturbances in obesity versus sleep apnoea: the importance of visceral obesity and insulin resistance. J Intern Med. (2003) 254:32–44. doi: 10.1046/j.1365-2796.2003.01177.x, PMID: 12823641

[59] Sanapo L Bublitz MH Bai A Mehta N Messerlian GM Catalano P. Association between sleep disordered breathing in early pregnancy and glucose metabolism. Sleep. (2022) 45:zsab281. doi: 10.1093/sleep/zsab281, PMID: 34999843 PMC8996028

[60] Llanos OL Galiatsatos P Guzmán-Vélez E Patil SP Smith PL Magnuson T. Pharyngeal collapsibility during sleep is elevated in insulin-resistant females with morbid obesity. Eur Respir J. (2016) 47:1718–26. doi: 10.1183/13993003.00918-2015, PMID: 27103392 PMC5937533

[61] Lévy P Kohler M McNicholas WT. Obstructive sleep apnoea syndrome. Nat Rev Dis Primers. (2015) 1:15015. doi: 10.1038/nrdp.2015.15 27188535

[62] Li M Chi X Wang Y Setrerrahmane S Xie W Xu H. Trends in insulin resistance: insights into mechanisms and therapeutic strategy. Signal Transduct Target Ther. (2022) 7:216. doi: 10.1038/s41392-022-01073-0, PMID: 35794109 PMC9259665

[63] Harada Y Oga T Chihara Y Azuma M Murase K Toyama Y. Differences in associations between visceral fat accumulation and obstructive sleep apnea by sex. Ann Am Thorac Soc. (2014) 11:383–91. doi: 10.1513/AnnalsATS.201306-182OC, PMID: 24471804

[64] Fang Y Su J Zhang B Zhao C Ji L Liang F. Autoantibodies of inflammatory cytokines as serum biomarkers in OSA patients. Clin Chim Acta. (2023) 547:117399. doi: 10.1016/j.cca.2023.117399, PMID: 37217113

[65] Redline S Azarbarzin A Peker Y. Obstructive sleep apnoea heterogeneity and cardiovascular disease. Nat Rev Cardiol. (2023) 20:560–73. doi: 10.1038/s41569-023-00846-6 36899115

[66] Szmyd B Rogut M Białasiewicz P Gabryelska A. The impact of glucocorticoids and statins on sleep quality. Sleep Med Rev. (2021) 55:101380. doi: 10.1016/j.smrv.2020.101380, PMID: 33010620

[67] Zhou T Chen S Mao J Zhu P Yu X Lin R. Association between obstructive sleep apnea and visceral adiposity index and lipid accumulation product: NHANES 2015-2018. Lipids Health Dis. (2024) 23:100. doi: 10.1186/s12944-024-02081-5, PMID: 38600516 PMC11005189

[68] McMillan A Morrell MJ. Sleep disordered breathing at the extremes of age: the elderly. Breathe (Sheff). (2016) 12:50–60. doi: 10.1183/20734735.003216, PMID: 27064674 PMC4818236

[69] Morrell MJ Finn L McMillan A Peppard PE. The impact of ageing and sex on the association between sleepiness and sleep disordered breathing. Eur Respir J. (2012) 40:386–93. doi: 10.1183/09031936.00177411 22241742 PMC3608395

[70] Fietze I Laharnar N Obst A Ewert R Felix SB Garcia C. Prevalence and association analysis of obstructive sleep apnea with gender and age differences - results of SHIP-trend. J Sleep Res. (2019) 28:e12770. doi: 10.1111/jsr.12770, PMID: 30272383

[71] Peppard PE Young T Palta M Dempsey J Skatrud J. Longitudinal study of moderate weight change and sleep-disordered breathing. JAMA. (2000) 284:3015–21. doi: 10.1001/jama.284.23.3015, PMID: 11122588

[72] Akanbi MO Agaba PA Ozoh OB Ocheke AN Gimba ZM Ukoli CO. Obesity and obstructive sleep apnea risk among Nigerians. J Med Trop. (2017) 19:110–5. doi: 10.4103/jomt.jomt_17_17, PMID: 29177137 PMC5701752

[73] Wang Y Mao L Zhang X. Waist-hip ratio is an independent predictor of moderate-to-severe OSA in nonobese males: a cross-sectional study. BMC Pulm Med. (2022) 22:151. doi: 10.1186/s12890-022-01886-3, PMID: 35459124 PMC9034636

[74] Zhao Y Yan X Liang C Wang L Zhang H Yu H. Incorporating neck circumference or neck-to-height ratio into the GOAL questionnaire to better detect and describe obstructive sleep apnea with application to clinical decisions. Front Neurosci. (2022) 16:1014948. doi: 10.3389/fnins.2022.1014948, PMID: 36312007 PMC9599743

[75] Ersözlü T Deniz M Fazlıoglu N Gultekin E Altintas N. Understanding potential associations between anatomic and other factors in OSA severity. Sleep Breath. (2022) 26:1649–53. doi: 10.1007/s11325-021-02539-1 34841491

[76] Dudley KA Patel SR. Disparities and genetic risk factors in obstructive sleep apnea. Sleep Med. (2016) 18:96–102. doi: 10.1016/j.sleep.2015.01.015, PMID: 26428843 PMC4602395

[77] Suri TM Ghosh T Mittal S Hadda V Madan K Mohan A. Systematic review and meta-analysis of the prevalence of obstructive sleep apnea in Indian adults. Sleep Med Rev. (2023) 71:101829. doi: 10.1016/j.smrv.2023.101829, PMID: 37517357

[78] Arnardottir ES Bjornsdottir E Olafsdottir KA Benediktsdottir B Gislason T. Obstructive sleep apnoea in the general population: highly prevalent but minimal symptoms. Eur Respir J. (2016) 47:194–202. doi: 10.1183/13993003.01148-2015 26541533

[79] Guerrero-Romero F Simental-Mendía LE González-Ortiz M. The product of triglycerides and glucose, a simple measure of insulin sensitivity. Comparison with the euglycemic-hyperinsulinemic clamp. J Clin Endocrinol Metab. (2010) 95:3347–51. doi: 10.1210/jc.2010-0288, PMID: 20484475

[80] Bastard JP Lavoie ME Messier V Prud'homme D Rabasa-Lhoret R. Evaluation of two new surrogate indices including parameters not using insulin to assess insulin sensitivity/resistance in non-diabetic postmenopausal women: a MONET group study. Diabetes Metab. (2012) 38:258–63. doi: 10.1016/j.diabet.2012.01.004 22405724

[81] Ding J Chen X Bao K Yang J Liu N Huang W. Assessing different anthropometric indices and their optimal cutoffs for prediction of type 2 diabetes and impaired fasting glucose in Asians: the Jinchang cohort study. J Diabetes. (2020) 12:372–84. doi: 10.1111/1753-0407.13000 31642584

[82] Young T Skatrud J Peppard PE. Risk factors for obstructive sleep apnea in adults. JAMA. (2004) 291:2013–6. doi: 10.1001/jama.291.16.2013 15113821

