# Supplementary material for: Association between triglyceride glucose-body mass index and obstructive sleep apnea: a study from NHANES 2015–2018
Source: Front Nutr. 2024 Aug 16;11:1424881. doi: 10.3389/fnut.2024.1424881 (PMC11363548; doi:10.3389/fnut.2024.1424881)
Supplement: Supplementary file 1 [file Table_1.pdf]

Supplementary Table 1 Stepwise screening of collinearity of independent variables

Covariates	β	*P*-value	VIF
Gender	-0.3073	<0.0001	1.3
Age	0.0100	<0.0001	1.3
TyG-BMI	0.4803	<0.0001	2.9
Race			1.3
Mexican American(Ref)	-	-	
Other Hispanic	-0.0501	0.6625	
Non-Hispanic White	-0.1691	0.0628	
Non-Hispanic Black	-0.121	0.2196	
Other Race	-0.2523	0.0147	
Edu level			1.3
Less than high school(Ref)	-	-	
High school	-0.1324	0.1344	
More than high	-0.1119	0.1338	
Smoking status	0.3333	<0.0001	1.1
Alcohol use	-0.0344	0.664	1.1
Diabetes	0.4946	<0.0001	1.3
Hypertension	0.4346	<0.0001	1.3
PIR	-0.0124	0.5268	1.3
CVD	0.3325	0.0003	1.2

β = beta (regression coefficients);VIFs = Variance Inflation Factors.

PIR, Poverty Income Ratio; CVD, Cardiovascular Disease;

TyG-BMI, triglyceride glucose-body mass index;

Supplementary Table 2 Association between BMI index and risk for OSA

Exposure	Model 1 OR(95%CI) P-value	Model 2 OR(95%CI) P-value	Model 3 OR(95%CI) P-value
BMI index	1.07 (1.06, 1.08) <0.0001	1.07 (1.06, 1.08) <0.0001	1.06 (1.05, 1.08) <0.0001
BMI quartile			
Q1	Reference	Reference	Reference
Q2	1.53 (1.29, 1.81) <0.0001	1.44 (1.22, 1.72) <0.0001	1.50 (1.22, 1.84) 0.0001
Q3	2.49 (2.10, 2.95) <0.0001	2.40 (2.01, 2.85) <0.0001	2.45 (1.97, 3.03) <0.0001
Q4	3.40 (2.86, 4.04) <0.0001	3.58 (3.00, 4.29) <0.0001	3.42 (2.75, 4.25) <0.0001

BMI, body mass index; TyG-BMI, triglyceride glucose-body mass index; OSA, obstructive sleep apnea;

Model 1: non-adjusted.

Model 2: adjusted for age, gender, and race.

Model 3: adjusted for age, gender, race, education level, PIR, smoking status, alcohol use, hypertension, diabetes, CVD

Supplementary Table 3 Association between TyG index and risk for OSA

Exposure	Model 1<br>OR(95%CI) P-value	Model 2<br>OR(95%CI) P-value	Model 3<br>OR(95%CI) P-value
TyG index	1.48 (1.36, 1.61) <0.0001	1.41 (1.29, 1.55) <0.0001	1.18 (1.05, 1.32) 0.0068
TyG index quartile			
Q1	Reference	Reference	Reference
Q2	1.37 (1.16, 1.61) 0.0002	1.28 (1.08, 1.51) 0.0048	1.19 (0.97, 1.47) 0.1009
Q3	1.76 (1.49, 2.07) <0.0001	1.63 (1.37, 1.93) <0.0001	1.36 (1.09, 1.69) 0.0057
Q4	1.98 (1.68, 2.34) <0.0001	1.80 (1.51, 2.14) <0.0001	1.26 (1.00, 1.58) 0.0492

TyG, triglyceride-glucose; OSA, obstructive sleep apnea;

Model 1: non-adjusted.

Model 2: adjusted for age, gender, and race.

Model 3: adjusted for age, gender, race, education level, PIR, smoking status, alcohol use, hypertension, diabetes, CVD
